# The ontogeny of mouse salivary gland macrophages is distinct between genders

**DOI:** 10.21203/rs.3.rs-5903499/v1

**Published:** 2025-02-06

**Authors:** Qingguo Zhao, Juhi Jaiswal, Ethan Chang, Arash Shahsavari, Yu Zhang, Victor Yu, Ronald Zheng, Fei Liu

**Affiliations:** 1 Cell Biology and Genetics Department, College of Medicine, Texas A&M University Health Science Center, College Station, TX 77843, USA.

**Keywords:** Salivary glands, macrophages, gender differences, lineage tracing, monocytes

## Abstract

Sexual dimorphism is found in gene expression and polarization of macrophages in mammals but remains unclear in the ontogeny of tissue-resident macrophages. Salivary glands show remarkable gender differences and macrophages here are essential for the maintenance of immune quiescence and tissue repair after damages. Therefore, we compared origins of macrophages in salivary glands between male and female mice using conditional Cx3cr1 and Ccr2 lineage tracing approaches. We found most salivary gland macrophages in adult males are locally maintained and derived from yolk sac progenitors or postnatal monocytes, whereas these cells in adult females are mostly short-lived and continuously replenished by monocytes. Female salivary glands also contain more monocytes and polarized macrophages. Cx3cr1 knockout made the macrophage ontogeny in adult male salivary glands like females. These gender differences appear related to female-dominant salivary gland phenotypes in a common autoimmune disease, Sjogren’s syndrome, and need be considered in developing macrophage-targeting therapies of dry mouth caused by autoimmunity or radiation.

## 1-Sentence Summary

In contrast to locally maintained male salivary gland macrophages, these cells in adult females are mostly short-lived and continuously replenished by monocytes, which appears related to female-dominant salivary gland phenotypes in Sjogren’s syndrome and need be considered in developing macrophage-targeting therapies of dry mouth.

## Introduction

In mammals, infectious diseases are more frequent and severe in males, whereas autoimmune diseases are more prevalent in females, indicating the sexual dimorphism in the immune system ^1, 2^. At the transcriptome level, such a dimorphism is mostly evident in macrophages than all other immune cells in mice ^3^. A related dimorphism of macrophage polarization and function has been reported in several tissues. In naive peritoneal and pleural cavities of mice and rats, the numbers of resident leukocytes are higher in females than in males, comprising more T and B lymphocytes and macrophages ^4^. These female resident macrophages exhibit more M1-like activities than males, whereas macrophage-derived cytokine production is diminished by proportionally more resident immunomodulatory CD4^+^ T lymphocytes ^4^. Conversely, in mouse models of viral myocarditis and asthma, macrophages in heart or lungs of males are more prone to M1 polarization, whereas those cells in females are more prone to M2 polarization, which leads to the sex-related susceptibility to these diseases ^5, 6^. These paradoxical reports suggest that the gender differences in macrophage polarization might be context- and/or tissue-specific. During steady state, many adult tissues in mammals contain both long-lived macrophages with prenatal origins and short-lived macrophages constantly replenished by adult monocytes, and the anatomic locations and functions of these two types of macrophages are distinct. For instances, in brain, lungs and skin, microglia, alveolar macrophages, and Langerhans cells are long-lived, whereas many neuron-associated macrophages, lung interstitial macrophages, and dermal macrophages are constantly replenished by adult monocytes ^7^. However, it remains unclear whether the ontogeny of tissue-resident macrophages in a same organ is comparable or different between genders. Answering this question will help us understand paradoxical gender differences of macrophage activities in different tissues and provide guidance in developing gender-optimized strategies for macrophage-targeted immune modulation.

In human, substantial gender differences have been noticed in biochemistry and flow of saliva ^8, 9^ and in gene expression in both minor and major salivary glands (SGs) ^10, 11^. Moreover, the autoimmune disease affecting SGs, Sjogren’s syndrome, occurs predominately in women over men (16:1) ^12^. In rodents, SGs also show significant gender differences in histology and gene expression ^13^, and female salivary glands are much more frequently affected than males in mouse models of Sjogren’s syndrome ^14^, suggesting that mouse models can help to better understand sex differences of immune responses in human SGs. Macrophages are abundant in healthy SGs ^15, 16^, related to the high sensitivity of SGs to radiation damage, and essential for function restoration of SGs through paracrine interactions with epithelial progenitors and endothelial cells ^17, 18^. The study on the origins of adult SG resident macrophages is limited and not conclusive ^19^, and it remains unclear whether such origins are comparable or different between genders.

Among the three pairs of major salivary glands, submandibular glands (SMGs) are the largest in mice and produce the highest volume of saliva. Therefore, we compared the origin and dynamics of SMG macrophages in male and female mice side-by-side with genetic lineage tracing, flow cytometry analysis, and gene knockout experiments. Our data indicate that most macrophages in adult male SMGs are locally maintained and derived from yolk sac progenitors or postnatal monocytes, whereas most macrophages in adult female SMGs are short-lived and continuously replenished by adult monocytes. Consistently, female mouse SMGs contain more monocytes and polarized macrophages, whereas *Cx3cr1* knockout made the macrophage ontogeny in adult male SMGs like that in females. These findings deepen our understanding of the salivary gland homeostasis and will help developing therapies targeting macrophages to restore salivary function damaged by radiation or autoimmune responses.

## Results

### 1. Yolk sac progenitors are a major source of macrophages in adult male SMGs but not in female SMGs.

**Figure 1.**
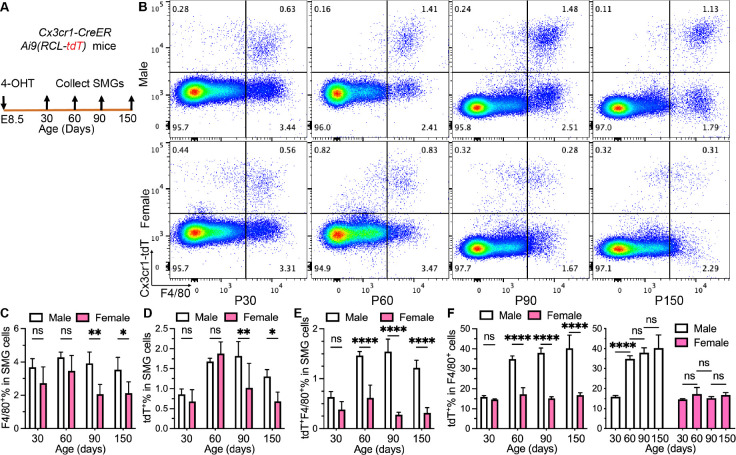
Lineage tracing of yolk sac progenitors in mouse SMG macrophages. A: Male and female *Cx3cr1-CreER;Ai9(RCL-tdT)* mice were induced at E8.5, and their SMGs were collected at P30, 60, 90 and 150 for flow cytometry analyses of F4/80 and tdTomato (tdT). B: Representative pseudo color plots of flow cytometry for tdT and F4/80 in all SMG cells. C-E: Percentages of F4/80^+^, tdT^+^, and tdT^+^F4/80^+^ in SMG cells were compared between males and females. F: Percentages of tdT^+^ cells in F4/80^+^ macrophages were compared between males and females or ages, respectively. N = 4. ns: not significant, *: p < 0.05, **: p < 0.01, ***: p < 0.001, ****: p < 0.0001.

Yolk sac macrophage progenitors are Cx3cr1^+^ and traffic to the embryo from E8.5 till E14.5 ^20^, therefore we used heterozygous *Cx3cr1-CreER;Ai9(RCL-tdT)* mice induced with 4-hydroxy-tamoxifen (4-OHT) at E8.5 (Fig. 1A) to trace progeny of yolk sac progenitors in SMGs as reported ^21^. This CreER system efficiently and comparably labeled microglial cells, the known yolk sac progeny, at both genders as reported ^22^ at postnatal day (P) 60 (Fig. S1A-C). In these mice, percentages of F4/80^+^ macrophages and tdT^+^ cells in SMG cells were comparable between genders at P30 and P60 but lower in females at P90 and P150 (Fig. 1B–D). At P30, tdT^+^F4/80^+^% in SMG cells and tdT^+^% in F4/80^+^ macrophages (~15%) were also comparable between males and females (Fig. 1E–F), indicating the similar initial labeling efficiency of this CreER system in SMGs between genders. At P60, percentages of tdT^+^ cells in F4/80^+^ macrophages in male SMGs increased to about 35% and maintained at P90 and P150. This index did not significantly change in female SMGs through P30 to 150 and was much lower than that in males from P60 to 150 (Fig. 1F). These data indicated that macrophages derived from yolk sac progenitors are long-lived in SMGs of both genders but much more abundant in adult males.

To determine whether there are differences in surface markers between yolk sac derived SMG macrophages and other macrophages in either gender, we examined the expression of tissue resident macrophages (TRM) marker Cx3cr1 ^23^, infiltrating macrophage marker Ccr2 ^24^, and pro-inflammatory macrophage marker Csf2rb ^25, 26^ in SMGs from P90 *Cx3cr1-CreER;Ai9* mice induced at E8.5 with flow cytometry. The majorities of both tdT^+^F4/80^+^ and tdT^−^F4/80^+^ cells in both genders are Cx3cr1^+^, Ccr2^−^, and Csf2rb^−^; but tdT^−^F4/80^+^ cells contain much more Cx3cr1^−^ cells, Ccr2^+^ cells, and Csf2rb^+^ cells in both genders (Fig. S1D-F). Notably, percentages of cells positive for either of these three markers in tdT^−^F4/80^+^ cells are significantly higher in females than males (Fig. S1G-H). These data indicated that in adult SMGs almost all tdT^+^F4/80^+^ cells are TRMs, whereas tdT^−^F4/80^+^ cells contain both TRMs and infiltrating macrophages recently derived from monocytes, and adult female SMGs contain more infiltrating macrophages than males.

### 2. Most SMG macrophages derived from fetal monocytes are short-lived in both genders.

**Figure 2.**
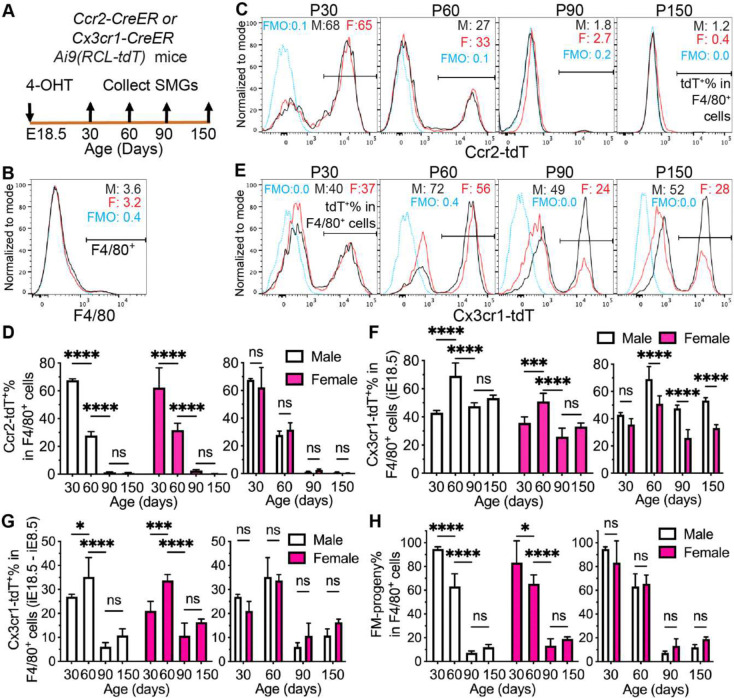
Lineage tracing of fetal monocytes in adult male and female SMG macrophages. A: Male and female *Ccr2-CreER;Ai9* or *Cx3cr1-CreER;Ai9* mice were induced at E18.5, and their SMGs were collected at P30, 60, 90 and 150 for flow cytometry analyses. B: F4/80^+^ cells in SMG cells were gated for following analyses. FMO, Fluorescence Minus One control; M, males; F, females. C-F: Percentages of Ccr2-tdT^+^ cells (C-D) or Cx3cr1-tdT^+^ cells (E-F) in F4/80^+^ macrophages were compared between ages or genders in mice induced at E18.5, respectively. G: Differences of Cx3cr1-tdT^+^% in F4/80^+^ macrophages between mice induced at E18.5 and E8.5 were compared between ages or genders, respectively. H: Percentages of total progeny of fetal monocytes (FM) were estimated and compared between ages or genders, respectively. N = 4. ns: not significant, *: p < 0.05, **: p < 0.01, ***: p < 0.001, ****: p < 0.0001.

Circulating fetal monocytes (FMs) are mostly (~90%) Ccr2^+^ and give rise to adult tissue-resident macrophages in multiple organs ^27^. Therefore, we firstly used *Ccr2-CreER;Ai9(RCL-tdT)* mice induced with 4-OHT at E18.5 to trace progeny of Ccr2^+^ fetal monocytes in SMGs from mice at P30, 60, 90 and 150. At P30, ~60% of F4/80^+^ macrophages in SMGs of both genders were labeled with Ccr2-CreER mediated expression of tdT (Ccr2-tdT, Fig. 2A–D), indicating that Ccr2^+^ fetal monocytes are a dominant source of macrophages in juvenile SMGs. However, Ccr2-tdT^+^% in F4/80^+^ macrophages decreased gradually in both male and females SMGs to about 30% by P60 and <3% at P90 and P150 (Fig. 2A–D), indicating that **al**most all SMG macrophages derived from Ccr2^+^ fetal monocytes are short-lived in both genders.

A small subset of circulating FMs is Ccr2^−^, and Ccr2^+^ FMs begin to decrease Ccr2 expression but increase Cx3cr1 expression upon tissue infiltration ^27^. Therefore, *Ccr2-CreER;Ai9* mice induced at E18.5 appears insufficient to trace all FMs. To address this issue, we induced *Cx3cr1-CreER;Ai9* mice with 4-OHT at E18.5 and subtracted the E18.5 induction data with the E8.5 induction data at the same age to estimate the Cx3cr1^*+*^ FM progeny in SMG macrophages. At P30, Cx3cr1-tdT^+^% and tdT^+^F4/80^+^% in SMG cells and Cx3cr1-tdT^+^% in F4/80^+^ macrophages are all comparable between males and females (Fig. S2D-F and Fig. 2E–F), indicating the similar initial labeling efficiency between genders. Cx3cr1-tdT^+^% in F4/80^+^ macrophages increased to around 70% in males and 50% in females at P60, decreased to around 50% in males and 25% in females at P90, maintained at these levels at P150, and are significantly higher in males than females from P60 to 150 (Fig. 2E–F and Fig. S2D). The percentages of Cx3cr1^+^ FM progeny in SMG macrophages can be estimated by subtracting tdT^+^% in F4/80^+^ cells in *Cx3cr1-CreER;Ai9* mice induced at E18.5 with that induced at E8.5. This indexes were ~30% at P60 in both genders and greatly decreased to ~10–15% at P90 and P150 without significant differences between genders at all ages examined (Fig. 2G). These data suggest that Cx3cr1^*+*^ fetal monocytes are also a major source of macrophages in juvenile SMGs, but only a small subset of their progeny is long-lived in adult SMGs of both genders.

The percentages of total FM progeny in SMG macrophages can be estimated by adding tdT^+^% in F4/80^+^ cells in Fig. 2D and 2G. From P30 to P150, this index is also dramatically decreased in both genders from ~90% to ~7% in males and ~13% in females (Fig. 2H). These data suggested that the progeny of fetal monocytes is dominant in SMG macrophages of juvenile mice but most of them are short-lived in both genders.

### 3. SMG macrophages derived from postnatal monocytes are short-lived in females but long-lived in males.

**Figure 3.**
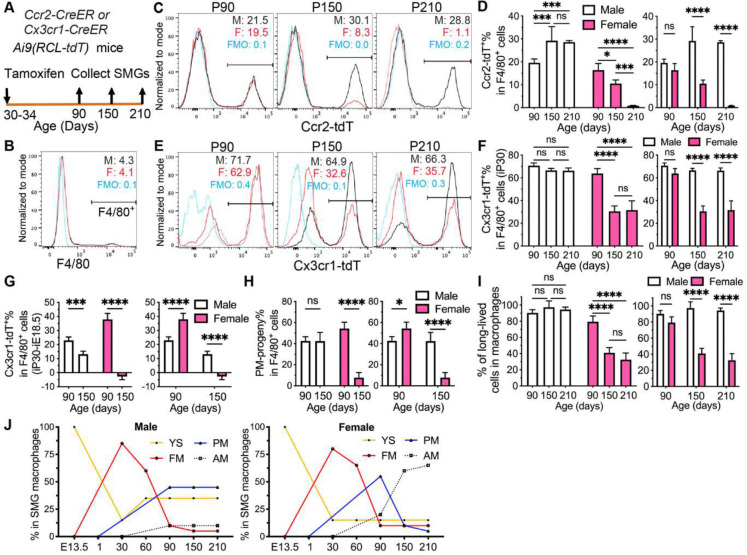
Lineage tracing of postnatal progenitors of SMG macrophages in both genders. A: Male and female *Ccr2-CreER;Ai9* or *Cx3cr1-CreER;Ai9* mice were induced at P30, and their SMGs were collected at P90, 150 and 210 for flow cytometry analyses of F4/80 and tdT. B: F4/80^+^ cells were gated for following analyses. C-F: Percentages of Ccr2-tdT^+^ (C-D) or Cx3cr1-tdT^+^ cells in F4/80^+^ macrophages (E-F) in mice induced at P30 were compared between ages or genders, respectively. G: Differences of Cx3cr1-tdT^+^% in F4/80^+^ macrophages in mice induced at P30 vs. E18.5 were compared between ages or genders, respectively. H: Percentages of total progeny of postnatal monocytes (PM) were estimated and compared between ages or genders, respectively. I: Percentages of long-lived cells in F4/80^+^ macrophages were compared between ages or genders, respectively. J: The dynamic contributions of 4 types of progenitors in SMG macrophages of male and female mice were estimated based on our above data and references. Yolk sac (YS) progeny was estimated in Fig. 1F–G. Fetal monocytes (FM) progeny was estimated in Fig. 2H. Since the YS data were stable from P60 to P150 and the FM data were negligible at P150, both data at P210 data were assumed comparable to P150. Postnatal monocytes (PM) data were estimated in Fig. 3H. Based on assumptions of YS and FM data at P210 and data in Fig. 3D and 3F, male PM data at P210 were assumed comparable to P150, whereas female PM data at P210 were assumed negligible. Adult monocytes (AM) data were estimated by subtracting 100% with data in Fig. 3I. N = 4. ns: not significant, *: p < 0.05, **: p < 0.01, ***: p < 0.001, ****: p < 0.0001.

Most monocytes in postnatal mice (~90%) are Ccr2^+^ classical monocytes and contribute to adult tissue-resident macrophages in some organs such as intestines ^28^. Notably, neonates have higher mean monocyte counts than adults, and postnatal monocytes (**PM**) are more pro-inflammatory than adult monocytes (**AM**), which is related to differences in DNA methylation and higher mortality of influenza infection in pediatric patients than adults ^29, 30, 31^. The immune responses in postnatal SGs are also different from adults, and children are more prone to SG infections like mumps ^32^. Because macrophages derived from Ccr2^+^ fetal monocytes are short-lived and negligible in SMGs by P90 (Fig. 2A–D), it is plausible to trace progeny of juvenile monocytes at and after P90 in *Ccr2-CreER;Ai9* mice induced with tamoxifen at P30. At P90, ~20% F4/80^+^ macrophages in SMGs of both genders were Ccr2-tdT^+^ (Fig. 3A–D), indicating that Ccr2^+^ juvenile monocytes initially contribute to SMG macrophages in both genders at comparable levels. At P150 and 210, Ccr2-tdT^+^% in SMG F4/80^+^ macrophages decreased gradually in females to about 3% but increased and maintained in males at about 30% (Fig. 3A–D), indicating that SMG macrophages derived from Ccr2^+^ juvenile monocytes (JM) are short-lived in females but long-lived in males.

However, some postnatal monocytes are Ccr2^−^, and some macrophages derived from Ccr2^+^ postnatal monocytes lose Ccr2 expression and begin to express Cx3cr1 in multiple mouse organs ^33^. The progeny of these cells in SMGs cannot be labeled in *Ccr2-CreER;Ai9* mice induced at P30. Therefore, we induced *Cx3cr1-CreER;Ai9* mice at P30 to trace these postnatal (P1 to P30) monocytes in addition to Cx3cr1^+^ macrophages derived from all fetal progenitors. The latter population has been traced in *Cx3cr1-CreER;Ai9* mice induced at E18.5 (Fig. 2), and the subtraction of the E18.5 induction data from the P30 induction data at the same endpoint could estimate the contribution of progeny of Cx3cr1^+^ postnatal monocytes. At P90, Cx3cr1-tdT^+^% in F4/80^+^ SMG macrophages were ~70% in males and ~50% in females induced at P30 (Fig. 3E–F), which were much higher (~20% in males and ~40% in females) than mice induced at E18.5 (Fig. 3G), suggesting that many macrophages in young adults of both genders are derived from Ccr2^−^Cx3cr1^+^ progeny of postnatal monocytes. At P150, Cx3cr1-tdT^+^% in macrophages maintained in males induced at P30 without significant changes, but the difference to mice induced at E18.5 significantly decreased. Meanwhile, this index in P150 females induced at P30 decreased greatly, and the difference to mice induced at E18.5 was negligible and significantly lower than that in males (Fig. 3E–G). At P210, Cx3cr1-tdT^+^% in SMG macrophages in each gender remained comparable with corresponding P150 data and lower in females than in males (Fig. 3E–F). The total percentages of postnatal monocytes (PM) progeny in SMG macrophages can be estimated as the sum of data in Fig. 3D and 3G. From P90 to P150, this index is also stable at ~40% in males but dramatically decreased in females from ~50% to ~8% (Fig. 3H). These data suggested that both Ccr2^−^Cx3cr1^+^ and Ccr2^+^ PM are a major source of adult SMG macrophages in male mice but not in females.

In mice induced at P30 and older than P90, percentages of long-lived cells in macrophages could be estimated as the sum of Ccr2-tdT^+^ and Cx3cr1-tdT^+^ macrophages (Fig. 3D and 3F) colonized SMGs before adulthood. This index was stable around 90% in males but significantly decreased and much lower in females than males at P150 and P210 (Fig. 3I). Conversely, macrophages negative for both tdT could be considered as short-lived macrophages continually replenished by adult monocytes (**AM**), which are much higher in females than males at P150 and P210 (~65% vs. ~10%). Inspired by the heterogeneity of TRM ontogeny in other adult tissues and the initial colonization of macrophages in SMGs at E13.5 ^28, 34^, the dynamic contributions of 4 types of progenitors (YS, FM, AM, and PM) to SMG macrophages of male and female mice were estimated based on data from Figures 1–3 (Fig. 3J). Collectively, these lineage tracing data suggested that most SMG macrophages are short-lived in females but long-lived in males, and much more adult female SMG macrophages are continually replenished by adult monocytes.

### 4. Female adult SMGs contain much more monocytes and pro-inflammatory macrophages derived from them than males.

**Figure 4.**
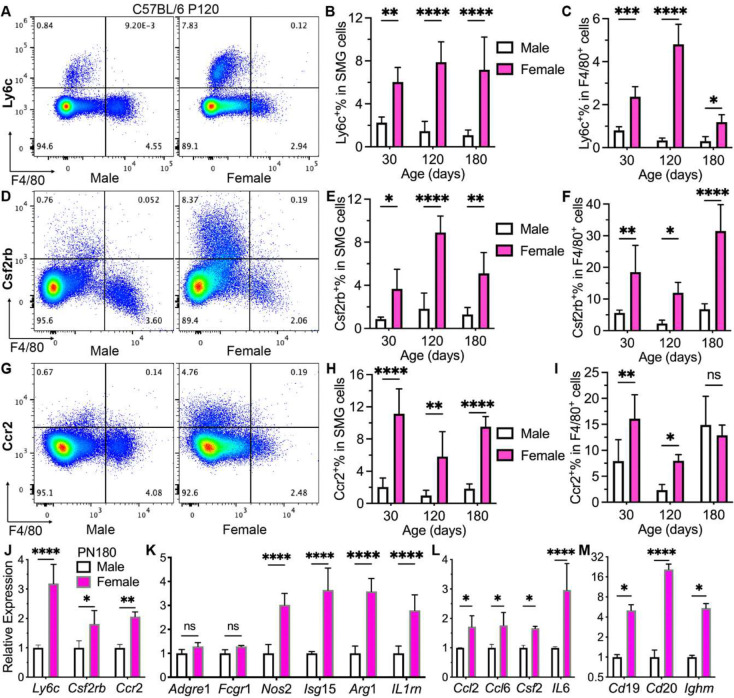
Gender differences in monocytes, monocyte-derived macrophages, and related gene expression in SMGs from wildtype mice. A-I: SMGs were collected from wildtype male and female C57BL/6 mice at P30, 120 and 180 for flow cytometry analyses of F4/80 and inflammatory classical monocyte markers Ly6c (A-C), Csf2rb (D-F), and Ccr2 (G-I). Representative pseudo color plots of P120 SMGs were shown in A, D, and G. Percentages of cells positive for these markers in SMG cells were quantified in B, E, and H. Percentages of F4/80^+^ macrophages positive for these markers in macrophages were quantified in C, F, and I. J-M: SMGs from male and female P180 C57BL/6 mice were examined with qRT-PCR for marker of monocytes (J) or macrophages and their polarization (K), related pro-inflammatory cytokines (L), and B cell markers (M). N = 4. ns: not significant, *: p < 0.05, **: p < 0.01, ***: p < 0.001, ****: p < 0.0001.

Above data from genetically modified lineage tracing mouse strains suggested that adult female SMGs contain more macrophages continuously replenished by monocytes than males during homeostasis. To validate that in wildtype mice, we examined the expression of macrophage marker F4/80 and multiple leukocyte/monocyte markers in SMGs from male and female C57BL/6 mice at P30, 120 and 180 with flow cytometry. Ly6c and Ccr2 are widely used markers of classical monocytes in mice ^24^, whereas Csf2rb is required for GM-CSF signaling essential for the inflammatory signature of Ccr2^+^Ly6c^+^ monocytes ^25^ and M1 polarization of macrophages ^26^. The expression of these markers in macrophages derived from classical monocytes is generally transient in various other tissues ^35^. Therefore, cells co-expressing F4/80 and Ly6c or Ccr2 are considered as macrophages derived from newly recruited monocytes, whereas cells co-expressing F4/80 and Csf2rb are considered as macrophages with higher pro-inflammatory potentials ^26^. In SMGs of C57BL/6 mice, percentages of cells positive for all these three markers are significantly higher in females than males at all three ages examined (Fig. 4A–I and Fig. S4A-C). Consistent to data in *Cx3cr1-CreER;Ai9* and *Ccr2-CreER;Ai9* mice, percentages of F4/80^+^ cells in SMG cells are comparable between genders at P30 but significantly higher in males than females at P120 and P180 (Fig.S4D). In F4/80^+^ SMG macrophages, percentages of Ly6c^+^, Csf2rb^+^, or Ccr2^+^ cells are also significantly higher in females than males at most ages examined except Ccr2^+^% at P180 (Fig. 4A–I and Fig. S4A-C). The higher expression of these 3 markers in female SMGs were validated with qRT-PCR assay of P180 mice (Fig. 4J). Moreover, qRT-PCR assay indicated that the expression levels of pan-macrophage markers *Adgre1* (F4/80) and *Fcgr1* are comparable between genders, but levels of both M1 markers (*Nos2 and Isg15*) and M2 markers (*Arg1 and IL1rn*) are much higher in females than in males (Fig. 4K), suggesting stronger polarization of macrophages in female SMGs. The expression of several related immune-modulatory cytokines, e.g. *Ccl2, Ccl6, Csf2,* and *IL6*, was also significantly higher in female SMGs than males (Fig. 4L). Since monocytes can also differentiated into dendritic cells and attract other immune cells into tissues, we compared expression of these immune cell markers in SMGs between genders by qRT-PCR. Levels of B cell markers, *Cd19, Cd20,* and *Ighm*, are dramatically higher in females than males (Fig. 4M, 5–20 folds), whereas markers for T cells (*Cd3d & Cd4*), innate lymphoid cells (*Ncr1 & NK1.1*), and dendritic cells (*Flt3*) are also higher in females to a lesser extent (Fig. S4E, 2–4 folds). However, mRNA levels of many other immune modulatory cytokines are comparable between male and female SMGs (Fig. S4F), possibly due to the enhanced feedbacks of more resident immunomodulatory cells in females as reported in peritoneal and pleural cavities ^4^. These data confirmed our lineage tracing data that female adult SMGs contain much more classical monocytes and macrophages recently derived from them than males. Moreover, these data suggest that some macrophage-related pro-inflammatory cytokines and multiple other types of immune cells are also more abundant in female SMGs, which likely contributes to the much higher penetrance of sialadenitis in females in mouse models of Sjogren’s syndrome.

### 5. *Cx3cr1* knockout compromised local maintenance of macrophages and increased monocytes and monocyte-derived macrophages in male but not female SMGs.

**Figure 5.**
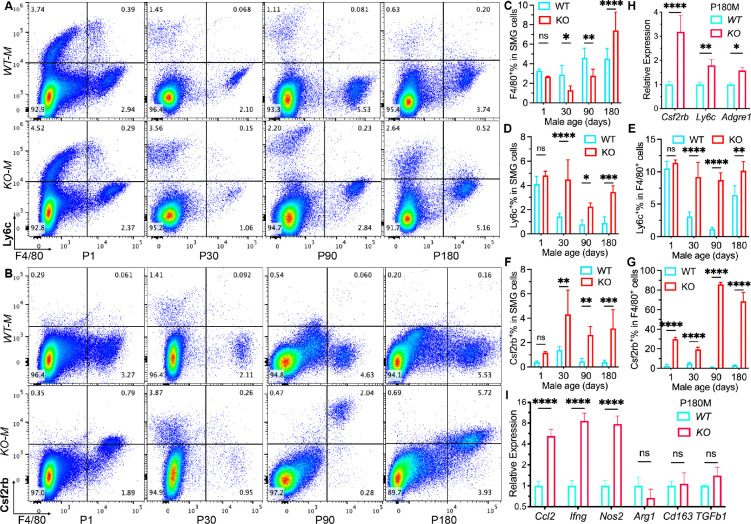
Effects of *Cx3cr1* knockout on relative numbers of macrophages, monocytes, and monocyte-derived pro-inflammatory macrophages in male SMGs. A-B: Representative pseudo color plots of flow cytometric analyses of Ly6c vs. F4/80 (A) and Csf2rb vs. F4/80 (B) in SMG cells from male wildtype (WT) and Cx3cr1 knockout (KO) mice at P1, 30, 90, and 180. C-G: Percentages of F4/80^+^, Ly6c^+^, and Csf2rb^+^ cells in SMGs and Ly6c^+^ or Csf2rb^+^ cells in F4/80^+^ cells were compared between male WT and KO mice at P1, 30, 90, and 180. H-I: SMGs from male P180 WT and KO mice were examined with qRT-PCR for markers of monocytes and macrophages (H) and their polarization (I). N = 4. ns: not significant, *: p < 0.05, **: p < 0.01, ***: p < 0.001, ****: p < 0.0001.

Cx_3_cr1 is highly expressed in yolk sac progenitors, anti-inflammatory nonclassical monocytes, and tissue resident macrophages including those in SGs ^17, 36^. Besides the chemotaxis function, Cx3cr1 signaling mediates maintenance of microglia and neuron-associated macrophages ^37, 38^. Interestingly, the sex-specific microglial responses to high-fat diet feeding in mice are also mediated by Cx3cr1 signaling, whereas *Cx3cr1* knockout female mice developed ‘male-like’ hypothalamic microglial accumulation and activation with increased susceptibility to diet-induced obesity ^39^. To determine roles of Cx_3_cr1 in the homeostasis of SG macrophages, we knocked out *Cx*_*3*_*cr1* by breeding homozygous *Cx*_*3*_*cr1-CreER* mice as reported ^40^. SMGs were collected from both male and female wildtype (WT) and *Cx*_*3*_*cr1* knockout (KO) mice at P1, 30, 90, and 180 for flow cytometry analysis of F4/80, Ly6c, and Csf2rb. In both males and females, the relative number of F4/80^+^ macrophages in SMGs was not significantly affected by *Cx*_*3*_*cr1* depletion at P1 (newborn) (Fig. 5A–C and S5), which is consistent to the report that the migration of macrophage progenitors from the yolk sac into several other tissues is independent of Cx_3_cr1 ^20^. However, this index was decreased at P30 and P90 but increased at P180 by *Cx*_*3*_*cr1* depletion in males (Fig. 5A–C). Meanwhile, *Cx*_*3*_*cr1* depletion significantly increased cells expressing Ly6c or Csf2rb in SMGs and relative numbers of Ly6c^+^ or Csf2rb^+^ cells in F4/80^+^ SMG macrophages of P60, 120 and 180 males (Fig. 5A–G). Notably, Csf2rb^+^ macrophages are marginal in WT male mice but significantly increased in KO mice since P1 and became dominant at P90 and P180 with peak Csf2rb expression at P90 (Fig. 5B and 5G). In females, *Cx*_*3*_*cr1* KO did not significantly affect most of these indexes but greatly increased Csf2rb^+^% in SMG cells at P180 and Csf2rb^+^% in macrophages at all ages (Fig. S5). The upregulation of *Csf2rb, Ly6c,* and *F4/80* (*Adgre1)* by *Cx3cr1*-KO in SMGs from male P180 mice were validated with qRT-PCR (Fig. 5H). Moreover, the expression levels of M1 markers (*Ccl2, Ifng, Nos2*) are much higher in KO than WT P180 males, whereas those of M2 markers (*Arg1, Cd163, Tgfb1*) are comparable between WT and KO (Fig. 5I), suggesting enhanced M1 polarization of macrophages in KO SMGs. These data suggested that although Cx3cr1 is not essential for the colonization of macrophages into SMGs at birth, it is required for the local maintenance of macrophages in adult male SMGs. Cx3cr1 depletion led to the replacement of these locally maintained macrophages by monocyte-derived macrophages with higher pro-inflammatory potentials in male SMGs, which is partially like the scenario in female SMGs. Consequently, by P180 *Cx3cr1* knockout significantly decreased saliva secretion in some male mice but not in females, which was accompanied with dramatic increases of mRNA levels of markers for T cells, B cells, innate lymphoid cells, and dendritic cells (Fig. S6A-C). However, we did not find the leukocyte foci typical in female mice with Sjogren’s syndrome in male *Cx3cr1* knockout mice with decreased saliva flow (Fig. S6D-G), suggesting that the *Cx3cr1* knockout alone leads to some but not all features of Sjogren’s syndrome in male SMGs.

## Discussion

Our lineage tracing data in *Cx3cr1-CreER;Ai9* mice induced at E8.5 showed that yolk sac progenitors are a major source of macrophages in adult male SMGs but not in female SMGs. The female data are consistent with the data from *Cdh5Cre-ERT2;Rosa26CAG-LSLtdT* mice induced at E7.5 ^19^. Since the gender of mice for the latter data was not specified, it remains unclear whether the male data from these two models are discrepant. As indicated by our lineage tracing data in *Cx3cr1-CreER;Ai9* and *Ccr2-CreER;Ai9* mice induced at E18.5 and P30, most macrophages are long-lived in male SMGs but short-lived in female SMGs, despite of their origins. The relative numbers of F4/80^+^ macrophages are lower in female SMGs than males since P90, and short-lived macrophages in adult female SMGs are continuously replenished by monocytes. Consistently, the relative numbers of cells positive for monocyte marker Ly6c or Ccr2 are much higher in female SMGs than male since P30. Moreover, *Cx3cr1* knockout made the ontogeny of macrophages in male SMGs “female-like”, increased expression of markers of leukocytes, and decreased saliva secretion in aged males. These data collectively indicate that the origins and dynamics of macrophages are distinct between genders in mouse SMGs.

The mechanisms underlying gender differences in SG macrophages need further exploration. In adult *C57BL/6* mice, sex-enriched expression of immune response genes was found in all major SGs, and multiple genes related to immune activation, such as JunB, are enriched in female SMGs ^13^. In human, the expression of anti-inflammatory genes, such as TGFβ2 and CFI, is much higher in male minor SGs than in females ^10^, whereas genes related to Response to wounding, such as the pro-inflammatory factor SPP1, are enriched in female parotids ^11^. In both mice and human, X-chromosome carries multiple genes regulating homeostasis and functions of macrophages, including their differentiation from hematopoietic stem cell (*IL3RA, GATA1*), polarization (*IL13RA*), activation (*CYBB*), and pattern recognition (*TLR7, TLR8, and IRAK1)* ^41^. Moreover, X-chromosome encodes much more microRNAs than any autosomes, and many X-linked microRNAs regulate immune responses ^42^. Although most differences in X chromosome dosage between sexes are mitigated through X chromosome inactivation (XCI), many genes evade XCI and remain expressed, referred to as XCI escape genes, in immune cells including macrophages in a tissue-specific manner ^43^. Y-chromosome carries much fewer genes but also plays an indispensable role in immune responses. The common loss of chromosome Y (LOY) in elderly men contributes to various diseases such as the increased risk of COVID progression by impacting granulocytes and monocytes ^44^, and LOY in leukocytes lead to dysregulation of ~500 autosomal genes ^45^. Besides these genes, sex hormones also play an important role in the gender difference of macrophage responses to TLR ligands: male hormone testosterone reduced the expression of TLR4 in macrophages and their sensitivity to TLR4 ligands, whereas the female hormone estradiol enhanced the expression of both TLR4, CD14 and inflammatory cytokines in macrophages ^46, 47^. Considering the prevalence of Sjogren’s syndrome in women over men ^12^ and the conserved X-chromosome genes and sex hormones regulating macrophages, it is likely that similar gender differences in SG macrophages are present in human.

In male SMGs, *Cx3cr1* knockout significantly decreased macrophages at P30 and P90 and dramatically increased the expression of Csf2rb in macrophages from P1. Similar compensatory induction of the CSF2Rb–STAT5 signaling axis has been reported in tumor associated macrophages upon CSF1R blockade ^48^. Therefore, our data suggested that Cx3cr1 signaling is required for the local expansion and/or maintenance of macrophages, whereas the depletion of *Cx3cr1* can be compensated by the induction of CSF2Rb signaling activity. In many tissues, Cx3cr1^lo^ macrophages are proinflammatory, whereas the Cx3cr1^hi^ macrophages are anti-inflammatory ^23, 49^. Conversely, Csf2rb signaling mediates M1 polarization of macrophages ^26^. Therefore, the induction of Csf2rb signaling by *Cx3cr1* knockout also enhanced inflammation in male SMGs.

One limitation of this study is the lack of the comparison of transcriptomes between male and female SMG macrophages. All published data on SG macrophages, including our own ^17, 36^, analyzed transcriptomes of macrophages from only male or female mice. Considering the huge gender differences mentioned above, it is necessary to compare transcriptomes of these cells between genders at both bulk and single cell levels in future studies. The other limitation is the lack of the comparison of responses of SG macrophages to radiation between genders. In SMGs of male mice, radiation acutely depleted proliferating macrophages and elevated macrophage replenishment from blood monocytes in long-term, and these macrophages are essential for epithelial regeneration after irradiation injury ^17, 19^. Since we found SMG macrophages in female mice are mostly short-lived and continuously replenished by monocytes, it is necessary to determine whether the responses and roles of macrophages in radiated female SGs are comparable to those in males.

## Conclusion

Macrophages in SMGs of adult male mice are mostly long-lived and maintained locally, whereas those in females are mostly short-lived, continuously replenished by monocytes, and more polarized. Correspondingly, steady-state female SMGs contain much more monocytes and other leukocytes than males, whereas the depletion of *Cx3cr1* made SMG macrophages in males partially “female-like”.

## Materials and Methods

### Animals

*Cx3cr1-CreER*^*T2*^, *Ccr2-CreER*^*T2*^, *Ai9(RCL-tdT),* and C57BL/6J mice were purchased from the Jackson Laboratory, housed in a specific-pathogen-free (SPF) animal facility managed by the Comparative Medicine Program of Texas A&M University, and acclimatized for at least 7 days before any experiments. All mouse procedures were approved by Texas A&M University IACUC. Sample size is based on our previous papers ^17, 36^ using the equation n=2SD^2^(z_α/2_+z_β_)^2^/Δ^2^ with Δ = 40% and SD = 20% to reach an acceptable power of 80% for statistics on numbers of SMG resident macrophages and expression levels of related genes. No criteria were set for including and excluding animals. SMGs from animals to be compared were collected within one hour and analyzed side-by-side by same investigators.

### Lineage tracing

Female 8–16 weeks old Ai9 mice were mated with male homozygous *Cx3cr1-CreER*^*T2*^*;Ai9(RCL-tdT)* or *Ccr2-CreER*^*T2*^*;Ai9(RCL-tdT)* mice. The morning that a vaginal plug was found was considered embryonic day 0.5. At embryonic day 8.5 (E8.5) and 18.5 (E18.5), these pregnant females were injected with 100 μg 4-hydroxytamoxifen (4-OHT, Sigma) intraperitoneally (IP) once. P30 *Cx3cr1-CreER*^*T2*^*;Ai9(RCL-tdT)* and *Ccr2-CreER*^*T2*^*;Ai9(RCL-tdT)* mice were injected with 100 μg 4-OHT IP daily for 3 days. At various ages, these mice were perfused with ~50 mL PBS to collect SMGs without blood immune cells for flow cytometry. For P1 mice, gender was determined by qRT-PCR of Y-chromosome gene Sry.

### Flow cytometry

Single cells from PBS-perfused mouse SMGs were generated as reported ^15^. Briefly, SMGs were minced and digested for 1 h with RPMI 1640 medium containing 2 mg/mL collagenase type I and 0.2 mg/mL DNase I (Gibco, Invitrogen) with continuous shaking at room temperature. The cell homogenate was filtered through 70-μm cell strainers and centrifuged at 350g for 3 minutes at 4°C to collect SMG cells. Cells were stained with two panels of three fluorescent labeled antibodies or corresponding Fluorescence Minus One (FMO) controls. Panel 1 included antibodies for F4/80 (BD Biosciences 569615), Ly6c (BD Biosciences 570264), and Csf2rb (Miltenyi Biotec 130–102-536). Panel 2 included antibodies for F4/80 (same as in Panel 1), Ccr2 (BD Biosciences 757169), and Cx3cr1(BioLegend 149007). Stained cells were analyzed on a CytoFlex flow cytometer (Beckman-Coulter). Cx3cr1-KO samples were analyzed side-by-side with WT samples at the same age. For all other flow cytometry assays, male and female samples at the same age were analyzed side-by-side. Data were analyzed with FlowJo 10.8.1 (FlowJo) software using gating strategy detailed in Supplementary Figure S1 as reported ^50, 51^.

### Quantitative reverse transcription polymerase chain reaction (qRT-PCR) assay

RNAs were extracted from SMGs with RNeasy Mini Kit (Qiagen) and reversely transcribed with High-Capacity cDNA Reverse Transcription Kit (Applied Biosystems). The qPCR was performed with SYBR Green Master Mix (Bio-Rad) on a CFX Opus 96 Real-Time PCR System (Bio-Rad). The primers were synthesized by Invitrogen with sequences retrieved from Primerbank (http://pga.mgh.harvard.edu/primerbank). The qPCR data were analyzed with *Gapdh* as the reference gene.

### Statistics

No animals or samples were excluded from the final analysis and samples were not pooled for analyses. The numbers of experimental replicates are 4 or 5, and technical replicates are 3 for qRT-PCR and 2 for flow cytometry. All statistical analysis and graphical generation of data were performed with Prism 10 software. Data from three or more groups were assessed using Two-way ANOVA with Bonferroni multiple comparisons test. Data from two groups were assessed using Student’s t-test.
